# The value of advanced MRI techniques in the assessment of cervical cancer: a review

**DOI:** 10.1007/s13244-017-0567-0

**Published:** 2017-08-21

**Authors:** Evelyn Dappa, Tania Elger, Annette Hasenburg, Christoph Düber, Marco J. Battista, Andreas M. Hötker

**Affiliations:** 1 Department of Diagnostic and Interventional Radiology, Johannes Gutenberg-University Medical Centre, Langenbeckstr. 1, 55131 Mainz, Germany; 2 Department of Gynaecology and Obstetrics, Johannes Gutenberg-University Medical Centre, Langenbeckstr. 1, 55131 Mainz, Germany

**Keywords:** Cervical cancer, DCE, DWI, IVIM, MRI

## Abstract

**Objectives:**

To assess the value of new magnetic resonance imaging (MRI) techniques in cervical cancer.

**Methods:**

We searched PubMed and MEDLINE and reviewed articles published from 1990 to 2016 to identify studies that used MRI techniques, such as diffusion weighted imaging (DWI), intravoxel incoherent motion (IVIM) and dynamic contrast enhancement (DCE) MRI, to assess parametric invasion, to detect lymph node metastases, tumour subtype and grading, and to detect and predict tumour recurrence.

**Results:**

Seventy-nine studies were included. The additional use of DWI improved the accuracy and sensitivity of the evaluation of parametrial extension. Most studies reported improved detection of nodal metastases. Functional MRI techniques have the potential to assess tumour subtypes and tumour grade differentiation, and they showed additional value in detecting and predicting treatment response. Limitations included a lack of technical standardisation, which limits reproducibility.

**Conclusions:**

New advanced MRI techniques allow improved analysis of tumour biology and the tumour microenvironment. They can improve TNM staging and show promise for tumour classification and for assessing the risk of tumour recurrence. They may be helpful for developing optimised and personalised therapy for patients with cervical cancer.

***Teaching points*:**

*• Conventional MRI plays a key role in the evaluation of cervical cancer.*

*• DWI improves tumour delineation and detection of nodal metastases in cervical cancer.*

*• Advanced MRI techniques show promise regarding histological grading and subtype differentiation.*

*• Tumour ADC is a potential biomarker for response to treatment.*

## Introduction

Cervical cancer remains the fourth most common cancer in women worldwide [1], showing particularly high incidence in countries with low socioeconomic status. There is wide regional variation in the use of imaging modalities, and magnetic resonance imaging (MRI) in particular, in the work-up of cervical cancer. Accordingly, the FIGO classification [2], which is the internationally recognised staging system for cervical cancer, relies solely on clinical examination in assessing tumour stage. However, the current FIGO classification acknowledges the use of imaging methods as an adjunct for cervical cancer staging, and a number of studies have shown that imaging, especially MRI, is superior to clinical examination alone for correctly evaluating cervical carcinoma stage [3–7]. This is of particular importance in regard to the identification of parametrial invasion and the correct assessment of tumour size, given their important implications for the choice of treatment, i.e. fertility-sparing surgery versus neoadjuvant chemotherapy [8, 9].

Lymph node status is not part of the FIGO classification system; however, lymph node metastases are an important independent adverse prognostic factor [10]. The depth of stromal tumour invasion is associated with an increased risk for lymph node metastases [11]. Surgical lymph node dissection remains the “gold standard” for the diagnosis of lymph node metastases, but it may be associated with postoperative complication rates of up to 17% [12] and with unwanted side effects such as lymphoceles and wound infections. Therefore, preoperative assessment of lymph node stage using imaging may have great clinical importance.

Histological subtype and grade of differentiation may also determine the course of the disease, the therapeutic outcome and patient survival [10, 13]. Squamous cell carcinomas represent the majority of cervical cancer cases (ca. 69%) compared to adenocarcinomas (ca. 25%) [14, 15]. Whether clinical outcome differs between squamous cell carcinomas and adenocarcinomas still remains controversial. However, rare tumour subtypes like neuroendocrine tumours have an unfavourable prognosis [16]. Low-grade tumours are associated with favourable outcomes and lower tumour recurrence rates compared to high-grade tumours [17]. Although histopathological samples can be acquired easily by biopsy prior to surgery, tumour heterogeneity may lead to sampling errors in large tumours [18, 19].

Where available, conventional MRI is the preferred imaging modality for evaluating the local extent of cervical cancer due to its excellent soft tissue contrast [20, 21]. Recently developed MRI techniques, namely diffusion weighted imaging (DWI) [22] and dynamic contrast enhanced MRI (DCE-MRI)—also termed multiparametric MRI—are already part of the standard MR work-up for other tumour entities [23]. These techniques also show promise as complementary techniques for the assessment of cervical cancer, as they allow the assessment of the tumour microenvironment rather than solely relying on conventional anatomical measurements (e.g. tumour size, infiltration of surrounding structures, etc.).

There is increasing interest and research effort focused on these new techniques. This systematic review summarises the current status of knowledge on the value of multiparametric MRI for the non-invasive assessment of parametrial invasion, lymph node status, tumour grading/subtype differentiation and response to chemotherapy, which are considered the most important clinical features for selecting a personalised therapeutic approach for an individual patient.

## Materials and methods

We adhered to the Preferred Reporting Items for Systematic Reviews and Meta-Analyses (PRISMA) Guidelines. A systematic literature search of PubMed and MEDLINE was conducted using the following search criteria: “MRI cervical cancer”, “DWI cervical cancer”, “DCE cervical cancer” and “IVIM cervical cancer”. The search was conducted on October 4, 2016, and it retrieved 2,393, 81, 66 and 6 references, respectively, for these searches. Only articles in English published in 1990–2016 were included. All articles were screened for relevance using the title and abstract. All study designs were eligible for inclusion. Studies on animals, duplicate studies and case reviews were excluded.

The resulting studies were reviewed independently by two authors (E.D. and A.M.H.). In the event of a disagreement about study inclusion, the two authors reached a consensus decision about inclusion after discussing whether the criteria for inclusion were fulfilled.

The primary eligibility criteria were that the articles reported on studies that examined the use of DWI, DCE-MRI or intravoxel incoherent motion (IVIM) for detecting parametrial invasion and lymph node metastases, determining tumour subtype and grading, and predicting tumour recurrence and therapeutic response.

The following data were extracted from full text articles: the study characteristics, including study design, year of publication, MRI sequences, histopathological findings and outcome(s). The final list of publications included 79 original research articles.

## Conventional MRI for staging cervical cancer

MRI has no role in evaluating stage IA cervical cancer, because microscopic disease cannot be reliably detected [20]. However, MRI is the preferred imaging method for tumours of stage higher than IB2, since it allows a highly accurate assessment of the extent of tumour infiltration. MRI is superior to clinical staging for evaluating tumour size and location [3–7], and it improves the accuracy of FIGO staging by up to 96% [7].

**Fig. 1:**
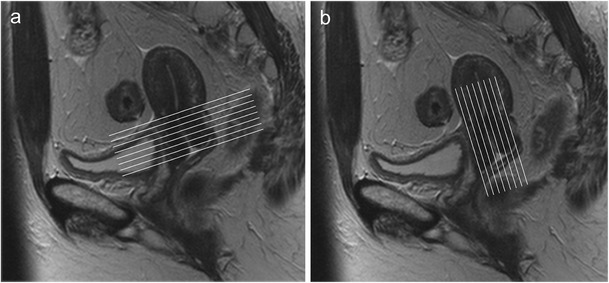
Sagittal T2-weighted image. The oblique axial (**a**) and coronal planes (**b**) are orientated to the plane of the cervical canal

The standard MRI protocol (Table 1) for cervical cancer includes T2-weighted imaging of the pelvis in different planes [20]. Axial and coronal planes are acquired obliquely, orientated to the plane of the cervical canal (Fig. 1). This allows for a more precise evaluation of tumour borders and parametrial invasion. T1- and T2-weighted sequences covering the whole abdomen and pelvis should be added to exclude identifiable lymph node metastases.

**Table 1:** Proposed MRI protocol for cervical cancer staging

**Sequence**		**Technical aspects**	**Comments**
T1 axial	TSE/2D GRE	Whole abdomen and pelvis ST 5 mm	Assessment of lymph nodes and distant metastases
T2 coronal	SS-TSE	Whole abdomen and pelvis ST 5 mm	Assessment of lymph nodes and distant metastases
T2 sagittal	TSE	Small FOV ca. 0.5 × 0.5 mm in-plane resolution ST 3 mm	Tumour evaluation (size, extension), assessment of rectal and bladder infiltration
T2 axial oblique	TSE	Small FOV ca. 0.5 × 0.5 mm in-plane resolution ST 3 mm perpendicular to long axis of cervical canal	Tumour extension, assessment of parametrial invasion and rectal and bladder infiltration
T2 coronal oblique	TSE	Small FOV ca. 0.5 × 0.5 mm in-plane resolution ST 3 mm parallel to long axis of cervical canal	Tumour extension, assessment of parametrial invasion in a second imaging plane
DWI axial oblique	EPI	Small FOV ST 3 mm *b* values 100, 600, 1,000 s/mm^2^	Tumour extension, assessment of parametrial invasion
Optional: multiphase 3D T1w fat-saturated sequences	GRE	ST 3 mm one native, four post-contrast scans	Alternatively DCE axial oblique (temporal resolution <10 s)

Preparation: fasting (>4 h), antiperistaltic agents, moderately filled bladder

*DCE* dynamic contrast enhanced, *DWI* diffusion weighted imaging, *EPI* echo-planar imaging, *FOV* field of view, *GRE* gradient echo, *ST* slice thickness, *TSE* turbo spin echo, *SS-TSE* single-shot turbo spin echo

## Advanced MRI techniques

**Fig. 2:**
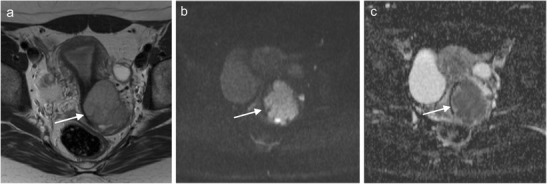
Axial T2-weighted image (**a**) and DWI image (*b* = 800) with ADC map (**c**) of a 36-year-old woman with stage IIB low-grade squamous cell cervical carcinoma. A hyperintense lesion is seen in the cervix (*arrow*), and there is disruption of the cervical stroma ring and parametrical invasion (**a**). The tumour shows high signal intensity on DWI (**b**), with corresponding low signal intensity on the ADC map (**c**)

DWI and DCE-MRI have emerged as promising tools for characterising uterine malignancies [20, 24]. DWI is based on the principles of Brownian movement of water molecules, and it gives information about tissue structure on a microscopic level [24, 25]. Since solid tumours generally exhibit an increased cell count, the diffusion of water molecules is restricted in tumour tissue. In contrast, diffusion is less restricted, for example, in areas of tumour necrosis, where water molecules can move more freely. To quantify these differences in tissues, special MRI pulse sequences are applied to measure the movement of the water molecules. Additionally, an apparent diffusion coefficient (ADC) map can be constructed, which allows for an easy visual assessment of the tumour (Fig. 2). In the literature, low ADC values have been shown to correlate with tumour aggressiveness in various tumour entities, such as prostate cancer [23, 26].

IVIM is an extension of DWI that allows the assessment of perfusion characteristics in tumourous tissue by visualising the microscopic motions of water molecules [27]. This allows the determination of several different parameters, namely: D, the true molecular diffusion coefficient; D*, the perfusion-related fast diffusion coefficient, which correlates with blood flow; and f, the perfusion fraction, which is linked to the blood volume within tissue [18, 27–29]. Neither DWI nor IVIM require the administration of contrast agents and can therefore be used in patients with renal insufficiency [24].

DCE-MRI is a dynamic examination technique that depicts changes in signal intensity over time after injection of a standard paramagnetic contrast agent. DCE-MRI is used to characterise tumour microcirculation. DCE-MRI can be evaluated either semiquantitatively to determine parameters that describe changes in signal intensity over time, or quantitatively, which requires the use of pharmacokinetic models. Quantitative measurements can be used to analyse perfusion and permeability parameters, such as the volume transfer constant between plasma and the extracellular extravascular space (K^trans^), which correlates with vascular permeability. DCE-MRI is already implemented in MR protocols that are used to evaluate other tumour entities, such as prostate carcinoma, to improve tumour detection in treatment-naive and post-treatment patients [30].

## Assessment of parametrial invasion

Conventional MRI has a reported diagnostic accuracy of 88.3–94%, a sensitivity of 38–100%, and a specificity of up to 92% in the assessment of parametrial invasion [31–34]. The signs of parametrial invasion on T2-weighted sequences include the disruption of the cervical stromal ring, spiculated tumour invasion and encasement of the periuterine vessels [35]. MRI can accurately exclude parametrial invasion, with a negative predictive value ranging from 94–100% [20, 36]. The intact hypointense stromal ring around the cervix on T2-weighted axial imaging represents the main sign for excluding parametrial invasion [37].

DWI further improves the assessment of parametrial invasion when it is added to conventional T2-weighted MRI [38–40]. Parametrial extension can be overestimated on T2-weighted images, especially in large tumours, which can induce changes in the surrounding stromal tissue due to tumour compression or increased inflammation [20, 41]. DWI reflects tumour cellularity, which helps differentiate between infiltrating tumour tissue and reactive changes. Park et al. [39] found that the fusion of DWI with T2-weighted images significantly (*p* < 0.05) increased diagnostic accuracy (reader 1, 90.1%; reader 2, 89.5%) compared to T2-weighted images alone (reader 1, 85.5%; reader 2, 83.6%). However, DWI is limited by poor anatomical detail and low spatial resolution; therefore, it should always be assessed in conjunction with T2-weighted images.

Studies have examined the correlation between low ADC values and tumour aggressiveness, including the presence of histopathological parametrial invasion [14, 42–44]. For example, Park et al. [44] found that patients with parametrial invasion had significantly lower tumour ADCs than those without and concluded that tumour ADC values are independent predictors of pathological parametrial invasion.

## Detection of lymph node metastases

Conventional MRI relies on the criterion of size for assessing pelvic lymph node involvement. Lymph nodes are rated as suspicious if the short axis diameter is greater 1 cm. However, given the overlap in size between metastatic, hyperplastic and normal lymph nodes and the fact that micrometastases in small lymph nodes are not uncommon [21], conventional MRI is limited in lymph node assessment, even when accounting for FIGO stage [45]. The literature shows that the diagnostic accuracy of MRI is 67–95%, its sensitivity is 37–90%, and its specificity is 71–100% [21, 32, 46–54], even when other signs of metastatic disease, such as loss of normal lymph node architecture, rounder form, irregular borders and heterogeneous signal intensity, are considered.

DWI may represent a powerful adjunct for differentiating between metastatic and non-metastatic lymph nodes, as it reflects differences in cellularity and histopathology between benign and malignant lymph nodes [55, 56]. Several studies have reported that lymph node metastases have a higher degree of diffusion restriction than normal lymph nodes, and this can be assessed quantitatively using ADC values [57–64]. Choi et al. [61] found that measuring the minimal ADC in a lymph node had greater sensitivity for detecting metastases than measuring the short-axis diameter (86% vs 55%, *p* < 0.001). Similarly, Liu et al. [57] reported a sensitivity of 95.7% and a specificity of 96.5% for the detection of metastatic nodes. Yet the results in the literature are conflicting [60, 65–67], as Rizzo et al. [67] found no significant association between DWI parameters and the presence of lymph node metastasis. However, the authors suggested that their results might have been influenced by patient selection (early clinical stage) and small patient number. Another study reported an overlap in mean ADC values between malignant lymph nodes and hyperplastic lymph nodes, which reduced the diagnostic accuracy to 78.4% [60]. However, the patients in that study were excluded if all of their lymph nodes had a short axis diameter under 5 mm, and this may have influenced the results [60]. Although a meta-analysis by Shen et al. [55] confirmed the usefulness of DWI for differentiation between benign and metastatic lymph nodes, study heterogeneity—especially regarding the lack of standard protocol for DWI—limits the comparability of these results. Further prospective studies with standardised MRI protocol may offer more evidence regarding the value of DWI for nodal detection.

Compared with PET/CT, routine MRI without DWI is less sensitive and accurate for the detection of nodal metastases [49, 54, 68]. A meta-analysis published in 2010 which included 41 studies found that PET and PET/CT had a higher diagnostic performance compared with MRI and CT [69]. The addition of DWI may increase the diagnostic performance of MRI compared with PET/CT. Monteil et al. [70] reported that MRI which included DWI sequences is more precise than FDG PET/CT for detecting pelvic lymph node metastases, with a sensitivity, specificity, and diagnostic accuracy of 67%, 84% and 81% compared to 33%, 92% and 81% for PET/CT; although the differences were not as pronounced for para-aortic lymph nodes. Kitajima et al. [71] also reported that DWI had higher sensitivity than FDG PET/CT (83.3% vs 38.9%) but lower specificity (51.2% vs 96.3%) for detecting lymph node metastases. Although these studies show that the addition of DWI clearly improves sensitivity of MRI for the detection of nodal metastases, PET/CT still has a higher specificity of up to 97% [69]. Especially in cases of advanced disease, PET/CT has a high sensitivity (75-100%) and specificity (87-100%) [20] and can help demonstrate sites of unexpected disease such as supraclavicular lymph nodes [72].

In the future, lymph node-specific MR contrast agents could further improve the assessment of lymph node metastases. There are new MR contrast agents that are classified as nanoparticles that contain ultrasmall particles of iron oxide (USPIO). These agents are taken up by macrophages into lymph nodes [73]. Metastatic lymph nodes displace macrophages, thereby preventing USPIO uptake. USPIO increases the sensitivity of MRI up to 93% for the prediction of nodal metastases [73, 74], and it can also improve tumour conspicuity [75, 76].

Currently, there are no USPIO contrast agents that are approved by the U.S. Food and Drug Administration (FDA) or European Medicines Agency (EMA) for clinical MRI applications [77]. Several first-generation USPIOs, such as SHU 555 C (Supravist) and AMI-227, were discontinued or are still awaiting approval. Ferumoxytol, a second-generation USPIO, is an FDA-approved drug that is used to treat iron insufficiency anaemia in patients with chronic kidney disease. Its application as an MRI contrast agent remains off-label, and it is no longer authorised for use in the European Union.

Another contrast agent, gadofosveset trisodium, which binds to human serum albumin and which accumulates in benign lymph nodes, has shown great value in precise lymph node staging in rectal cancer MRI [78]. Thus, it may also be of value in staging cervical cancer.

## Grading and subtype differentiation

The ADC has value in differentiating between normal uterine cervix stroma and cervical carcinoma [79–87]. Furthermore, the ADC shows potential for assessing pathological subtypes and for tumour grade differentiation [14, 60, 88–92]. A recent study [93] reported lower ADC values in poorly differentiated tumours compared to well/moderately differentiated tumours (*p* = 0.02). However, there were no significant differences between squamous cell carcinomas and adenocarcinomas (*p* = 0.1). In contrast, Xue et al. [92] retrospectively investigated ADC values in 53 patients with histopathologically proven cervical cancer and found a significant difference in the mean ADC values between adenocarcinomas and squamous cell carcinomas (*p* = 0.0074). However, there are currently no established ADC cutoff values as variations in MRI techniques and protocols can affect ADC values. In case of the previously mentioned studies, Winfield et al. [93] used a 3-T scanner with an endovaginal coil, resulting in higher signal-to-noise ratio and spatial resolution compared to a 1.5-T scanner with eight-channel phased-array body coil used in the study of Xue et al. This hinders comparisons between different centres and studies [94, 95].

In addition to DWI, IVIM may be useful for evaluating tumour differentiation and perfusion [18, 28, 93, 96]. IVIM perfusion parameters show moderate to good correlation with perfusion parameters derived from DCE-MRI in cervical cancer (*r* = 0.42–0.58; *p* = 0.003–0.038) [96]. Recently, mono-exponential DWI values (“classical” ADC values) were compared to IVIM model parameters in 42 cervical cancer patients, with both ADC and D (true molecular diffusion coefficient) showing lower values in poorly differentiated tumours than in well/moderately differentiated tumours [93]. To investigate differences between cancer tissue and normal cervical stroma, Lee et al. [28] compared the IVIM characteristics of 16 patients with cervical cancer with those of 17 healthy controls. Cervical cancer had lower perfusion and diffusion IVIM values compared to normal cervical tissue and leiomyoma tissue. Zhou et al. [18] reported higher perfusion at the tumour edge in high-grade tumours and found that D was significantly higher in G1 tumours than in G3 tumours. Moreover, the authors proposed that measuring tumour perfusion at the tumour edge might be better than measuring the whole tumour volume. Notably, during rapid tumour growth, cell proliferation often exceeds tumour angiogenesis, leading to poorly perfused central tumour areas and leading to tumour heterogeneity. Yamashita et al. [97] examined 62 cervical cancer patients using DCE-MRI. They found that areas with high contrast enhancement represented cancer cell fascicles and that poorly enhanced areas contained mainly fibrous tissue.

## Tumour recurrence and therapeutic response

Differentiating residual tumour tissue or tumour recurrence from post-therapeutic tissue changes, such as inflammation and fibrosis, remains challenging using conventional MRI, as both scar tissue and residual tumour tissue can have similar signal intensities. The ideal time to predict therapy response is not yet defined. Some authors advocate early assessment 2 weeks after radiochemotherapy [98]. In difficult cases, functional imaging, including DWI and DCE, can be a useful addition. Mahajan et al. [99] examined 30 patients after hysterectomy with suspected local tumour recurrence. Additional multiparametric sequences, including DWI and DCE, increased the diagnostic accuracy to 100% compared to 70% accuracy with conventional MRI alone. Similarly, another study reported that a combination of T2-weighted images and DWI had a higher diagnostic accuracy in the detection of post-treatment tumour recurrence versus T2-weighted images alone (92.1% vs 73.6%, *p* = 0.016) [100].

The tumour ADC has been described as a potential biomarker for response to treatment. As noted above, the tumour ADC can be used to assess tumour grading, which can reflect tumour aggressiveness [92] and which is associated with treatment resistance. Several authors have compared pre-treatment ADC values in patients with versus without later tumour recurrence, finding that low pre-treatment ADC values seem to be a strong predictor of later tumour recurrence [22, 101–107]. Furthermore, changes in ADC values during chemotherapy, radiotherapy or chemoradiation may help evaluate the treatment response [42, 43, 108–123]. For example, Kuang et al. [42] examined 75 patients prior to, during and after therapy completion and found significantly higher ADCs in patients with complete response compared to those with partial response or stable disease after therapy completion. Accordingly, the ADC seems to be a good biomarker for monitoring the early tumour response [112, 113, 115, 117, 119, 122, 124], as increasing ADC values reflect increased diffusivity, possibly due to treatment-induced necrosis, apoptosis-induced cell death, loss of cell membrane integrity and increased extracellular space [42, 124, 125].

DWI may also have value as a predictor of long-term disease control. A recent study examined post-treatment DWI in 100 patients 1 month after completion of chemoradiation [126]. They found that the presence of residual tumour tissue determined on post-treatment T2-weighted images with the addition of DWI improved the prediction of disease progression up to 3 years after treatment, with a positive predictive value of 72.7% compared to 39.3% for T2-weighted images without DWI.

IVIM techniques may also be useful for predicting and monitoring tumour response, with results that are comparable to mono-exponential ADC modelling [120, 127]. However, so far only a few studies have investigated IVIM in this context. Zhu et al. [127] examined 21 patients who were receiving chemoradiation therapy and showed an increase of both f (the perfusion fraction) and D* (the pseudoperfusion coefficient) in the first weeks of chemoradiation therapy.

The value of DCE-MRI in predicting tumour response is well studied. Tumours with low perfusion characteristics are associated with tumour hypoxia, which represents a negative prognostic factor in cervical cancer [128]. In contrast, more oxygenated tumours may be more sensitive to radiation and chemotherapy, leading to a better prognosis [129, 130]. DCE-MRI can be used to predict the treatment response in cervical cancer [97, 117, 129, 131–146] and can show longitudinal changes in tumour perfusion during treatment [132, 142]. Mayr et al. [142] demonstrated that persistent low perfusion prior to, during and after radiotherapy correlates with a high risk of treatment failure; in contrast, patients with higher perfusion prior to therapy or with therapy-induced increases in initially low perfusion show a better outcome. According to Halle et al. [134], DCE-MRI can identify patients with hypoxia-related chemoresistance by correlating hypoxia-related gene sets with a previously determined prognostic DCE-MRI parameter (A_Brix_). Early identification of tumours with persistent low perfusion and, consequently, a higher chance of treatment failure warrants further investigation, as this could drive a change in treatment strategy and help the field move toward a more personalised treatment approach.

## Limitations

There are limitations to DWI and DCE-MRI in that there are some technical issues and, most importantly, a lack of standardisation. The technical limitations include differences in modelling and the choice of *b* values for diffusion-weighted MRI, plus there are a large variety of pharmacokinetic models and sequences/contrast agent injection protocols used for DCE-MRI. Moreover, continuous technical advances and constant optimisation of MRI protocols according to the newest technical standards inadvertently reduce the comparability of MRI studies, as changes in resolution or the introduction of new advanced MRI techniques might allow for more precise tumour depiction. This had led to limited reproducibility and, since ADC values differ between various centres and with different MRI scanners, there are currently no established ADC cut-off values that allow for precise differentiation between, for example, tumour subtypes or grades [95]. Standardisation of MRI techniques is therefore considered critical for improving comparisons between studies.

Regarding the assessment of lymph node metastases, several studies excluded lymph nodes smaller than 5 mm, leading to selection bias. Node-by-node correlations between preoperative MRI and histopathological specimens present another problem. Lymph node position is generally labelled according to lymph node region, which limits the correlation between suspicious MRI findings and histopathological samples. In addition, the small size of some lymph nodes makes it difficult to precisely position a region of interest in order to measure, for example, ADC in a single lymph node.

## Conclusions

Conventional MRI plays a key role in the evaluation of cervical cancer, showing good results for the assessment of tumour extent and parametrial invasion. New techniques, such as DWI, IVIM and DCE, show promise as tools for viewing cervical tumours and for quantitative analysis of tumour biology and the microenvironment. The addition of DWI improves the determination of tumour extension and the detection of lymph node metastases. Both DWI and DCE might provide further insights into tumour biology in terms of histological grading and subtype differentiation, and thereby help to assess the risk of tumour recurrence. Large multicentre prospective studies are needed to determine whether these new techniques can be used to develop optimised and personalised therapies for patients with cervical cancer.
